# The Role of Response Bias in Perceptual Learning

**DOI:** 10.1037/xlm0000111

**Published:** 2015-04-13

**Authors:** Pete R. Jones, David R. Moore, Daniel E. Shub, Sygal Amitay

**Affiliations:** 1 Medical Research Council (MRC) Institute of Hearing Research, Nottingham, United Kingdom and University College London Institute of Ophthalmology; 2 Medical Research Council (MRC) Institute of Hearing Research and Cincinnati Children’s Hospital Medical Center, Cincinnati, Ohio; 3 School of Psychology, University of Nottingham; 4 Medical Research Council (MRC) Institute of Hearing Research

**Keywords:** bias, perceptual learning, signal detection theory

## Abstract

Sensory judgments improve with practice. Such *perceptual learning* is often thought to reflect an increase in perceptual sensitivity. However, it may also represent a decrease in response bias, with unpracticed observers acting in part on a priori hunches rather than sensory evidence. To examine whether this is the case, 55 observers practiced making a basic auditory judgment (yes/no amplitude-modulation detection or forced-choice frequency/amplitude discrimination) over multiple days. With all tasks, bias was present initially, but decreased with practice. Notably, this was the case even on supposedly “bias-free,” 2-alternative forced-choice, tasks. In those tasks, observers did not favor the same response throughout (stationary bias), but did favor whichever response had been correct on previous trials (nonstationary bias). Means of correcting for bias are described. When applied, these showed that at least 13% of perceptual learning on a forced-choice task was due to reduction in bias. In other situations, changes in bias were shown to obscure the true extent of learning, with changes in estimated sensitivity increasing once bias was corrected for. The possible causes of bias and the implications for our understanding of perceptual learning are discussed.

Observers’ sensory judgments often improve with practice (Wright & Fitzgerald, 2001; Fine & Jacobs, 2002). It is generally assumed that such *perceptual learning* reflects increased sensitivity to the task-relevant information, either through more efficient decision strategies (Gold, Sekuler, & Bennett, 2004; Jones, Moore, Shub, & Amitay, 2014) or decreased internal noise (Lu & Dosher, 2008; Jones, Shub, Moore, & Amitay, 2013). However, information extraction is only one step in the decision process—the observer must then compare the sensory evidence to a criterion in order to determine an appropriate response (see Figure 1). Ideally, this criterion should be placed so as to maximize some payoff metric, such as percent correct. In practice though, an observer’s criterion may deviate from the ideal, making one response more likely even when the sensory evidence supports another (see Figure 2). Thus, a biased observer may exhibit a systematic preference toward pressing a particular button, or giving a particular answer. Any such bias will affect performance, and changes in bias could in principle explain some or all perceptual learning. The present study evaluated this possibility by quantifying the extent to which bias is present in naïve observers (Experiments I and II), is reduced by practice (Experiments I and III), and can explain improvements in performance (Simulations).[Fig-anchor fig1][Fig-anchor fig2]

The role of bias has received little previous attention within the perceptual learning literature. This is for two main reasons. The first is practical. Learning studies often employ adaptive tracks, and/or use three or more response options. Such methods can make estimates of performance faster or more reliable (see Amitay, Irwin, Hawkey, Cowan, & Moore, 2006), but make computing bias metrics problematic. Multiple response options complicate matters, since every pair of responses may have its own corresponding bias, each of which must be estimated independently. Furthermore, in some multi-interval designs (e.g., odd-one-out) no models have yet been formulated for characterizing response bias (Macmillan & Creelman, 2005, pp. 235–251). Adaptive staircases also make bias more difficult to compute, because the ideal criterion depends on the expected magnitude of the signal (see Figure 2). Bias may therefore vary across trials, as the stimulus is adapted up or down. This would again require bias to be estimated multiple times, potentially resulting in a multitude of bias parameters too numerous to estimate reliably. In Experiment I, we therefore studied learning using a simple yes/no detection task in which there was only a single ideal criterion (Method of Constant Stimuli). Notably, this same approach has already been used recently to study the effects of yes/no bias on perceptual learning (Wenger & Rasche, 2006; Wenger, Copeland, Bittner, & Thomas, 2008). In those studies, it appeared that response bias actually increased with practice. Conversely, in the present study we use a novel method of analysis to show how the same behavior actually represents an overall reduction in bias.

The second reason why bias is often overlooked is theoretical. Learning effects are prevalent on *m*-alternative forced-choice [mAFC] tasks, and mAFC tasks are widely believed to preclude bias. If this assumption is correct, then it follows that learning must be independent of bias. However, the evidence that mAFC tasks preclude bias is incomplete. What is clear is that both the constant error term used in psychophysics (Gescheider, 1997), and metrics *c* and *logβ* used in signal detection theory (SDT; Macmillan & Creelman, 1990; Dusoir, 1975; Wickens, 2002), tend to be small on mAFC tasks and the values tend to vary little with practice (e.g., Schoups, Vogels, & Orban, 1995; Ben-David, Campeanu, Tremblay, & Alain, 2011; Campbell & Small, 1963). However, these measures only index a constant tendency to favor one response (stationary bias). In contrast, bias may also be nonstationary. It may fluctuate randomly; for example, if the observer is unable to maintain a stable criterion (e.g., as shown by Kubovy & Healy, 1977). Or it may vary systematically; for example, if the observer is influenced by the events of previous trials. Crucially, nonstationary biases are not obviously discouraged by mAFC designs—an “alternating” observer may be just as inclined to respond “Interval 1” after “Interval 2” as they are to respond “Yes” after “No”. Moreover, nonstationary biases cannot be detected using traditional bias measures, since these average over all trials (i.e., whereupon equal-and-opposite preferences for A after B and B after A will cancel out). Experiment II therefore analyzed trial-by-trial response dependencies to examine the extent to which nonstationary bias is present in naïve observers. Notably, previous studies using such techniques have tended to indicate that sequential dependencies are small in magnitude, and extend over only one or two trials (e.g., Jesteadt, Luce, & Green, 1977; see Experiment II for details). Here we replicate previous results, but also show that for a particular subset of trials, bias effects can be long lasting and substantial. Furthermore, in Experiment III we extend this result to perceptual learning, and examine the extent to which nonstationary biases decrease with practice.

Finally, significant changes in bias are not sufficient to judge effect size. Therefore, to assess the importance of bias in perceptual learning, observer responses were simulated with and without various forms of bias. On the basis of these data, bias-correction factors were derived by which true sensitivity can be recovered given estimates of performance and bias. By applying these corrections to the multisession learning data of Experiments I and III, the relationship between observed changes in bias and performance were quantified. Practice-induced changes in perceptual sensitivity were shown to be at times smaller, and at times greater, than would otherwise be apparent.

To summarize, reductions in bias can potentially explain the improvements in performance observed during perceptual learning. These response preferences can be constant (stationary bias) or vary depending on previous trials (nonstationary bias). Experiment I investigated whether stationary bias is present in naïve observers performing a yes/no task, and, if so, whether it decreases with practice. Experiments II and III extended this work to the more typical 2AFC paradigm. Here stationary bias was expected to be minimal, but nonstationary bias was expected to be present in naïve observers (Experiment II), and was expected to decrease with practice (Experiment III). Finally, we used simulations to relate the observed changes in bias to changes in threshold performance, and derived correction factors.

## General Method

Here we describe those methods that were common across all three experiments.

### Participants

Participants were normal hearing adults with no prior experience of auditory psychophysics. Normal hearing was assessed by audiometric screening, administered in accordance with the British Society of Audiology recommended procedure (≤20 dB HL bilaterally, at 0.5 kHz to 4 kHz octaves; British Society of Audiology, 2004). Participants were recruited through advertisements placed around Nottingham University campus, and received £7.5/h compensation. Experiments were conducted in accordance with Nottingham University Hospitals Research Ethics Committee approval and informed written consent was obtained from all participants.

### Stimuli Generation and Apparatus

Stimuli were digitally synthesized in Matlab v7.4 (2007, The MathWorks, Natick, MA) using a sampling rate of 22.05 kHz and 24-bit quantization, were converted to analog signals by a PCI sound card (Darla Echo; Echo Digital Audio Corporation, Carpinteria, CA), interfaced via the Psychophysics Toolbox v3 (Brainard, 1997) ASIO wrapper (Steinberg Media Technologies, Hamburg), and were presented diotically via Sennheiser headphones (Experiment I: HD25-II; Experiment II: HE60; Experiment III: HD480II). Observers were tested individually in a double-walled sound-attenuating booth; they had an unlimited time to respond using a button box, and received visual feedback via an LCD monitor.

## Experiment I: Stationary Bias on a Yes/No Task

The purpose of this experiment was to assess how much stationary bias (i.e., a constant preference toward one response) is present in naïve observers, and the degree to which stationary bias is reduced by practice.

The task was yes/no amplitude modulation detection. A yes/no decision paradigm was of particular interest for two reasons. First, because the use of yes/no tasks is widespread—particularly with animals, clinical groups, and other populations where test duration and memory limitations are concerns (see Green, 1993). Second, because it is the only paradigm in which the role of bias in perceptual learning has been previously examined (Wenger & Rasche, 2006; Wenger et al., 2008).

In the study by Wenger and Rasche (2006), observers practiced a yes/no visual contrast detection task, using sets of randomly interleaved stimulus levels (Method of Constant Stimuli). Those observers who improved with practice were found to become increasingly liberal (predisposed to say “yes”) when bias was evaluated at an arbitrary, fixed stimulus level. This would seem to suggest that perceptual learning actually leads to an increase in bias. Alternatively though, it may be that observers maintained a single response criterion, which they learned to optimize across all stimulus levels. In this case, overall global bias may have decreased with practice, at the cost of local bias increasing at some particular stimulus level(s). These two hypotheses were examined in the present experiment, using a measure of global bias in which the observers’ sensitivity at all stimulus levels was taken into account. The task was an auditory analog of the visual detection task used in Wenger and Rasche (2006).

### Method

Thirteen normal hearing observers (4 female; mean age 21.9) performed a one-interval, yes/no, sinusoidal amplitude modulation [SAM] detection task, in which the observer was asked “did the loudness of the sound fluctuate?”. Of these 13, one observer was excluded from all analyses due to an apparent loss of concentration—despite having the lowest detection threshold of all listeners in Session 1; by Session 7 there was no correlation between target and response [*r*_598_ = 0.06, *p* = .175], and no threshold could be estimated.

The stimuli were amplitude-modulated bandpass noises, similar to those used in Fitzgerald and Wright (2011). The carrier was a 3–4 kHz bandpassed Gaussian noise. The amplitude modulator was an 80 Hz sinusoid. As shown in Figure 3A, the modulation depth (or *index*) varied between 0 (*no modulation*) and 1 (*full modulation*), with the trial-by-trial value determined by the stimulus condition (see below). The stimuli were 400 ms in duration, including 10 ms *cos*^2^ on/off ramps, and were presented at an average level of 70 dB SPL in all conditions (adjusting for modulation depth).[Fig-anchor fig3]

Each trial commenced with a 300-ms warning interval, during which a visual fixation cross was displayed. This was followed by a single 400-ms stimulus observation. Participants were then given an unlimited time to respond, after which visual feedback was presented for 300 ms prior to the next trial onset.

Each session consisted of 600 randomly ordered trials (Method of Constant Stimuli), with short breaks after the 200th and 400th trial. Half (300) of the trials were noise trials (modulation depth = 0) and half were signal trials (0 < depth ≤1). The 300 signal trials consisted of 30 trials at each of 10 modulation depths, uniformly spaced between α and β on a logarithmic scale. In session one: α = .1 and β = 1. In subsequent sessions, α and β were set to the modulation depths required to attain 5% and 95% correct detection performance in the previous session, as estimated from cumulative Gaussian fits. This session-by-session adaptive procedure followed Wenger and Rasche (2006), and was designed to minimize floor/ceiling effects.

Participants completed seven sessions within 2 weeks, with no more than one session per day. Before the first session participants were given three examples of an unmodulated noise (depth = 0), and three examples of a fully modulated noise (depth = 1).

### Measures and Analysis

Performance was indexed by the 79% correct detection limen, DL_79_, which was derived from a cumulative Gaussian, fitted to hit rate as a function of log-modulation index. Psychometric fits were made using the psignifit toolbox (v2.5.6), which implements the maximum-likelihood procedure of Wichmann and Hill (2001).

Bias was measured in two ways. First, as per Wenger and Rasche (2006), local bias at a single signal level was assessed using the traditional SDT metric, *c* (or: λ_*center*_):

$$c=\lambda_{obs}-\lambda_{ideal}=-Z(f)-\frac{d\prime }{2}=-\frac{1}{2}[Z(f)+Z(h)],$$ 1

where λ_*ideal*_ is the ideal criterion, λ_*obs*_ is the observer’s criterion, *Z* is the inverse of the cumulative Gaussian distribution (i.e., Φ^−1^), and *f* and *h* denote false alarm and hit rates respectively. The metric *c* is calculated at a constant stimulus level—in this case the modulation depth requisite for a 79% hit rate in session one. This modulation depth was determined independently for each observer in session one. In each subsequent session sensitivity, *d*′, was estimated at that same modulation depth (interpolating between presented depths if required), and bias was assessed relative to an ideal criterion, λ_*ideal*_, equal to $\frac{1}{2}$ *d*′.

Global bias was estimated in the same manner, but after accounting for sensitivity across all 10 signal levels, thus:

$$\begin{array}{l} c_{global}=\lambda_{obs}-\lambda_{ideal}=-Z(f)-\underset{\lambda }{arg\;max}\left(\overset{m}{\underset{i=1}{\sum }}(P({\text{S}}_{i})[\Phi (\lambda ;\;d_{i}\prime ,1)]\right)\\ \;\;\;\;\;\;\;\;\;\;\;\;\;\;\;\;\;\;\;\;\;\;\;\;\;\;\;\;\;\;\;\;\;\;\;+P(\text{N})[\Phi (\lambda ;\;0,1)]), \end{array}$$ 2

where *d*_*i*_′ is the observer’s sensitivity to the *i*th stimulus level, *P*(S_*i*_) is the probability of the *i*th signal condition occurring $\left(\frac{1}{20}\right)$, and *P*(*N*) is the probability of a noise trial occurring $\left(\frac{1}{20}\right)$. Note that Equation 2 is a direct generalization of Equation 1, and the two equations differ only in how the ideal criterion, λ_*ideal*_, is computed. Specifically, while Equation 1 only considers performance at a single signal level, in Equation 2 the ideal criterion is that which maximizes performance over all stimulus conditions (i.e., taking into account the observer’s sensitivity to each signal, and the probability of each signal/noise occurring). If there was only one signal level then Equation 2 would be equivalent to Equation 1. For details on how Equation 2 was derived, see Section S1 (online Supplemental Material).

### Results

#### Learning

As shown in Figure 3B, performance improved across sessions, with more practiced observers able to detect significantly smaller amplitude modulations [*F*_(6,66)_ = 5.80, *p* < .001, η_*p*_^2^ = 0.35]. Ten individuals (83%) exhibited improvements, and the majority of learning occurred during the first session. These findings are consistent with those reported previously for this task (e.g., Fitzgerald & Wright, 2011). There was no consistent relationship between starting performance and amount of improvement [Spearman’s rho; *r*_10_ = −0.04, *p* = .921, *n*.*s*.].

#### Bias

As in Wenger and Rasche (2006), local bias, *c*, increased across sessions. Measured at a single signal level, observers appeared to be unbiased initially [*CI*_95*%*_ = −0.04, 0.20], but became progressively more liberal (prone to say “yes”) with practice [*F*_(6, 66)_ = 3.42, *p* = .005, η_*p*_^2^ = 0.24].

However, once all stimulus conditions were taken into account this pattern was reversed (Figure 3C). Group-mean global bias, *c*_*global*_, was initially liberal [*t*_11_ = −6.16, *p* < .001], but *decreased* across sessions [*F*_(6,66)_ = 5.11, *p* < .001, η_*p*_^2^ = 0.32]. By Session 3 (after 1,200 preceding trials) no significant global bias was present [*t*_11_ = −0.14, *p* = .888, *n*.*s*.]. At the individual level, 10 observers (83%) exhibited this pattern of bias magnitude reduction (though one of these individuals was initially conservative). The session-by-session changes in mean global-bias-magnitude correlated strongly with improvements in performance [*r*_5_ = −0.85, *p* = .017], suggesting that reductions in bias are related to improvements in detection thresholds.

### Discussion

Observers are often assumed to be unbiased agents, basing their responses only on the available sensory evidence. In a yes/no detection task, this assumption was shown to be acceptable only after 1,200 practice trials. In contrast, naïve observers exhibited significant (global) bias, generally in favor of responding “yes” (liberal). Reductions in bias correlated robustly with improvements in performance, suggesting that bias reduction is a substantive component of perceptual learning on a yes/no task.

This work is consistent with previous reports that observers can learn to adjust their criterion based on feedback (Herzog & Fahle, 1999; Herzog, Ewald, Hermens, & Fahle, 2006; Aberg & Herzog, 2012). Moreover, the results are consistent with data derived using an analogous visual task (Wenger & Rasche, 2006), though our ultimate conclusion is different. Thus, as in Wenger and Rasche (2006), local bias (i.e., bias measured at a single stimulus level) increased with practice. However, when all signal levels were considered, observers were shown to be becoming less biased overall, as per the ideal observer.

The fact that naïve observers tended to respond liberally may reflect a belief that incorrect misses (No|Signal) are more costly that incorrect hits (Yes|Noise). Alternatively, it may be that the initial tendency to say “yes” is driven by sensory factors. Thus, as detailed in the General Discussion, a liberal bias can also arise if the observer underestimates the amount of noise inherent in their sensory system. In either case, a similar bias toward responding “yes” would also be expected on other yes/no detection tasks. Accordingly, Wenger and Rasche (2006) also observed the same pattern of behavior on a visual contrast detection task, though we know of no systematic review of yes/no bias under Method of Constant Stimuli. Finally, it is important to note that the present findings would not necessarily be replicated using other methods. For example, when using an adaptive tracking procedure the observer can anticipate the next signal level, and so can vary their criterion from trial-to-trial. In those circumstances it remains to be seen whether yes/no bias is present in naïve observers, the direction of such biases, or whether it decreases with practice, although as discussed in the general introduction, measuring bias in more complex designs is often challenging.

## Experiment II: Nonstationary Bias on a 2AFC Task (Naïve Observers)

Experiment I demonstrated that bias decreases with practice on a yes/no task. However, many perceptual learning studies use forced-choice paradigms that are intended to preclude bias occurring in the first place. The purpose of Experiment II was to assess whether forced-choice tasks do preclude bias, by quantifying bias in naïve observers performing a 2AFC task. Effects of practice are detailed separately, in Experiment III.

As discussed in the Introduction, stationary bias is likely to be low in forced-choice tasks, but nonstationary bias—specifically, the tendency to favor one response depending on the events of the previous trials—may be more substantial. Indeed, that observers’ responses on forced-choice tasks are liable to be influenced by preceding trials has long been noted.^1^ For example, Green (1964) reported “a tendency among all observers to choose the interval opposite the one on which they had just been correct.”

Trial-by-trial response dependencies can be quantified using a variety of techniques. For example, Verplanck, Collier, and Cotton (1952) used a serial-correlation procedure (Wald & Wolfowitz, 1943) to assess the statistical independence of sequential luminance-detection responses, made when performance was near chance. Runs of identical responses were observed to be greater in length (and thus fewer in number) than would be expected if each response had been made independently. This implies that observers were biased toward repeating their previous response (hereafter *presponse*). Similar results have also been found using an information analytic approach Garner (1953) as well as through multiple regression (Jesteadt et al., 1977) and related auto-correlation techniques (Gold, Law, Connolly, & Bennur, 2008).

In the present experiment, nonstationary bias was measured in two ways. First, by using a multiple regression method, described previously by Jesteadt et al. (1977). Therein, presponses are used to predict which response occurred subsequently. If a significant proportion of response variability is explained by the presponses, then this indicates that trial-by-trial judgments were not made independently. Notably, this technique tends to indicate that response dependencies are small in magnitude and limited in range. For example, Jesteadt et al. (1977) found that 2.9% of variance on a loudness estimation task was explained by the immediate presponse, and that including longer runs of presponses did not significantly improve the power of the model. This suggests that response dependencies only extend over a single trial. Accordingly, recent behavioral works in ferrets (Alves-Pinto, Sollini, & Sumner, 2012), mice (Busse et al., 2011) and rhesus monkeys (Gold et al., 2008) have also found evidence of sequential shifts in response criterion, but these effects have again been limited primarily to the last preceding trial. Notably though, in the present work we predicted that for a subset of trials, levels of bias may be larger, more long lasting, and cumulative across trials. In particular, it was thought that runs of consistently identical responses (same answer, same result) would lead to strong biases to either perseverate if correct, or alternate if incorrect. This was assessed using a second, novel method of analysis in which the traditional SDT bias measure, *c*, was applied to independent subsets of data, depending on what the presponses had been and whether they were correct.

The task was 2AFC tone discrimination, in which observers had to judge which of two tones was greater in either frequency or intensity. Notably though, and unbeknown to the observer, both tones were identical, making the task impossible and the feedback arbitrary. Impossible tasks have been previously shown to induce learning (Amitay, Irwin, & Moore, 2006), and are well suited for examining bias, since expected sensitivity is guaranteed to be zero (and bias is liable to be underestimated as sensitivity increases; see Section S2 in the online Supplemental Material). Possible drawbacks to this approach are addressed in the Discussion, below.

### Method

Thirty observers (20 female; mean age 22.6) completed 500 trials of a two-interval, two-alternative, forced choice [2I2AFC], pure tone discrimination task, in which both tones were identical on every trial (impossible discrimination). Half (15) of the observers were instructed to “identify the higher tone,” while the other 15 were instructed to “identify the louder tone.” Regardless of the task instructions, both tones were 1 kHz sinusoids, 100 ms in duration, including 10 ms *cos*^2^ on/off ramps. The two tones were separated by a 500 ms interstimulus interval, and were presented diotically at 80 dB SPL.

Trial-by-trial feedback was presented visually for 500 ms after each response. Since the two tones were identical, the “correct” tone (for the purposes of scoring and feedback) was selected randomly. The ideal observer would thus be expected to perform at chance. For the present purposes correctness therefore relates primarily to whether observers believed that their presponse was correct. (*N.B*. observers were unaware when questioned subsequently that the feedback was arbitrary.)

### Measures and Analysis

Stationary bias was assessed using the forced-choice equivalent of Equation 1, thus:

$$c=\frac{\sqrt{2}}{2}[Z(P_{C〈NS〉})-Z(P_{C〈SN〉})],$$ 3

where *P*_*C*〈NS〉_ is the proportion of correct Interval 2 (noise-signal) responses, *P*_*C*〈SN〉_ is the corresponding proportion of correct Interval 1 (signal-noise) response. The $\sqrt{2}$ adjustment was simply to scale this 2AFC measure of *c* so as to make it comparable with *c* in the yes/no task in Experiment I.

As discussed in the Introduction, nonstationary bias was assessed in two ways. First, via multiple regression. Here, the identity, *I* (Interval 1 or 2), and correctness, *C* (true or false), of the previous *N* responses were used to predict the response identity on trial *t*, thus:

$$I_{t}=\left(\sum_{i=1}^{N}\alpha_{i}I_{t-i}+\beta_{i}C_{t-i}\right)+\gamma +\epsilon ,$$ 4

where α, β, and γ are the estimated regression coefficients, and ϵ is a Gaussian error term. This approach is identical to that reported previously by Jesteadt et al. (1977), with the following two exceptions. First, signal magnitude was not entered into the model, since all stimuli were identical (impossible discrimination). Second, we additionally entered the correctness of the preceding responses into the model, since observers were observed to respond differently if their presponse had been deemed “correct”/“incorrect” (e.g., see Table 2). Note, however, that since the stimuli were identical throughout, “correctness” was arbitrary and determined at random.[Table-anchor tbl2]

Nonstationary bias was also measured in a second, novel manner, by deriving a separate measure of bias, *c*, that depended on the events of the preceding trials. To do this, trials were classified by the pattern of previous responses (‘presponses’), and Equation 3 was applied independently to each of the resultant subsets. The principle difficulty with this approach is data sparseness. Even with only two variables (identity and the correctness), many patterns of presponses will be observed only once during the course of the experiment. This sparseness was mitigated in two ways. First, we made the Markov assumption that observers’ criterion, λ, was conditional only upon the last *N* presponses. When *N* = 0, bias was calculated with no regard for the preceding trials, as per the traditional SDT approach. When *N* = 1, bias was calculated using only those trials where the single preceding response was of a particular identity and correctness (e.g., where the presponse was Interval 2 and correct). As *N* increased, progressively more presponses were taken into account. Second, sparseness was further reduced by examining only runs of identical presponses (all same interval and correctness). It was speculated that such runs would affect observers most consistently, though other patterns of presponses may also induce biases. Thus, at each level of *N*, four measures of bias were derived: c|(‘Interval1’∩Correct),c|(‘Interval1’∩Incorrect),c|(‘Interval2’∩Correct),c|(‘Interval2’∩Incorrect).

Notably, trials preceded by *N* identical responses may also be preceded by *N* + 1 identical responses. This may lead to estimates of bias being artificially inflated at lower levels of *N*. Accordingly, when computing bias each trial was only evaluated once, at the highest possible value of *N* (where *N*_*max*_ = 3). This is illustrated in Table 1, which shows how a typical sequence of responses was subdivided to calculate c | (“*Interval2*”∩Correct) at various levels of *N*.[Table-anchor tbl1]

### Results

Group-mean stationary bias did not differ significantly from zero [*t*_29_ = 0.45, *p* = .656, *n*.*s*.]. This indicates that, unlike in the yes/no task of Experiment I, naïve observers did not have a consistent preference for one response alternative (Table 2; row 1).

To test for nonstationary bias, the regression model of Equation 4 was applied to each individual. The identity and correctness of the preceding response significantly predicted the subsequent responses in 19 of 30 observers [*p* < .05], and explained on average 3.3% of response variability. This indicated that most observers were influenced by their presponses, but that the effect was small. To examine whether sequential dependencies extended to longer runs, the number of presponses considered by the model was progressively increased. Including a second presponse explained, on average, an additional 1.2% of response variance, and a third presponse explained a further 0.7%. However, these improvements were not significant [both *p* ≥ .8, *n*.*s*.], suggesting that only the immediate presponse substantively affects observers’ decisions. There was substantial individual variability, however, and in one observer a second presponse improved *R*^2^ by 10%.

Nonstationary bias was then analyzed for a specific subset of trials by measuring bias, *c*, conditional on previous trials. Table 2 (rows 2–5) shows that observers tended to alternate after incorrect presponses, and perseverate after correct presponses.

This result is extended to longer presponse runs in Figure 4. As the number of identical and correct presponses increased, observers became progressively more biased toward repeating the same response (top-left panel). Thus, repeated Interval 1 responses were likely to be followed by a further Interval 1 response, while repeated Interval 2 responses were likely to be followed by a further Interval 2 response. To compare Interval 1 (bottom curve) and Interval 2 (top curve) presponses, the values of one were compared to the additive inverse of the other. A repeated-measures analysis of variance [rmANOVA] yielded no significant difference between these curves [*F*_(1,24)_ < 0.01, *p* = .966, *n*.*s*.], indicating that the strength of the perseverance effect was similar, regardless of whether the presponses had been Interval 1 or Interval 2.[Fig-anchor fig4]

Group-mean bias magnitudes, averaged across both presponse identities (bottom-left panel), consistently increased as *N* increased [rmANOVA: *F*_(3,87)_ **=** 14.33, *p* < .001, η_*p*_^2^ = 0.33]. However, there was significant variability between observers [*F*_(29,58)_ = 2.28, *p* = .004], with some observers exhibiting greater perseverance than others. This result is consistent with the individual data reported in Section S3 (online Supplemental Material).

For responses following incorrect presponses, the relationship between *N* and bias was nonmonotonic (top-right panel). After only one incorrect presponse (*N* = 1), responses were biased in favor of the alternate interval. However, after three identical, incorrect responses (*N* = 3), observers were inclined to perseverate. Again, mean bias magnitude (bottom-right) was found to increase as a function of *N* [rmANOVA: *F*_(3,87)_ **=** 14.30, *p* < .001, η_*p*_^2^ = 0.33]. Note that in this format, unlike with the signed values (top-right), substantial bias was observed in the *N* = 2 condition. This is because of cancellation between observers (i.e., at *N* = 2, some continued to alternate, while some began to perseverate).

Half (15) of the observers were instructed to perform a frequency discrimination, and half were instructed to perform an intensity discrimination. Since the task was impossible, the stimuli were the same in both cases (two identical tones). However, to investigate whether levels of bias were affected by the initial task instructions, mean bias magnitude was analyzed in a mixed-effects ANOVA, with *N* presponses as a within-subjects factor, and Instruction Type as a between-subjects factor (two levels: frequency discrimination; intensity discrimination). No significant difference was observed between the two groups [*F*_(1,28)_ = 2.10, *p* = .160, *n*.*s*.], indicating that the task instructions did not affect bias.

### Discussion

These data demonstrate that naïve observers are biased even on a 2AFC sensory judgment task. Although stationary bias was minimal, levels of nonstationary bias were substantial, with observers favoring whichever response had been correct on previous trials. Thus, responses were liable to repeat following positive feedback, and alternate following negative feedback.

That observers can be affected by sequential trial dependencies has been reported previously (e.g., Jesteadt et al., 1977; Gold et al., 2008). Notably though, the effects have tended to be small in both magnitude and duration. When a previous analysis technique was used in the present study, this pattern was replicated. The immediate presponse explained 3.3% of response variability (a value in good agreement with the 2.9% reported by Jesteadt et al., 1977), and only the single preceding trial affected most observers’ responses substantively. However, using a novel analysis method, sequential dependencies on a subset of responses were shown be long-lived and cumulative. Furthermore, these nonstationary biases were symmetric across response intervals (i.e., correct Interval 1 responses encouraged further Interval 1 responses, and correct Interval 2 responses encouraged further Interval 2 responses). Since traditional (“molar”) performance measures such as *d′* and percent correct average across all trials, these biases would not be apparent, and would instead manifest as lower sensitivity.

One concern with Experiment II is that the impossible nature of the task (identical tones) may have caused observers to behave unusually. Against this are the facts that (a) the observers were unaware when questioned that the task was impossible; and (b) the data are consistent with Jesteadt et al., 1977, where the task was not impossible. However, to address this possibility more directly, Experiment III applied the same techniques to judgments of suprathreshold (nonidentical) stimuli. This also allowed learning effects to be evaluated.

## Experiment III: Nonstationary Bias on a 2AFC Task (Learning)

Experiment II showed that nonstationary bias is present in unpracticed observers. Experiment III examined to what extent this bias decreases with practice on a 2AFC task. Unlike in Experiment II, the stimuli were not identical, allowing learning to be evaluated.

There is good evidence that sequential dependencies can be reduced with practice. This evidence is provided principally by studies of the gambler’s fallacy (Ayton & Fischer, 2004; Jarvik, 1951), and other related recency effects. For example, Lindman and Edwards (1961) constructed shuffled decks, equally composed of Red and Green cards. Observers were asked to predict the color of each card in turn. Alternation (or “negative recency”) was observed initially, with observers tending to avoid guessing the most recently occurring outcome (e.g., preferring to predict “Green” after a run of “Red” cards). Such alternation was reduced in the second half of the experiment, with observers tending toward chance in their responses (see also Edwards, 1961). This suggests that response dependencies can be modified through practice. However, it remains unclear whether these results—obtained using tasks where outcomes are predicted a priori—generalize to psychophysical tasks containing an actual signal, where judgments are made a posteriori, and where the use of information from previous trials is discouraged (often through explicit instruction). Furthermore, it remains unclear to what extent any such changes in response-dependencies can explain the improvements in performance commonly observed during perceptual learning. Accordingly, in Experiment III we examined to what extent nonstationary bias is reduced by practice on a quintessential perceptual learning task: pure tone frequency discrimination.

### Method

This dataset was a subset of that detailed previously in Amitay, Hawkey, and Moore (2005). Twelve observers (mean age 29.3; 7 female) performed seven blocks of frequency discrimination across four sessions (3,850 trials total). Each block consisted of 550 trials, with five interleaved tracks of 100 adaptive trials, and 50 randomly occurring catch trials in which the target interval was trivially apparent (50 Hz stimulus difference).

On each trial, observers were presented with two pure tones separated by a 500-ms interstimulus interval. Each tone was 100 ms in duration, including 20 ms *cos*^2^ on/off ramps, and was presented diotically at 70 dB SL. The test frequency was always greater-than or equal-to the standard tone frequency, which was fixed at 1 kHz. On adaptive trials, the frequency difference was determined by a two-down one-up transformed staircase (Levitt, 1971). The initial frequency difference, Δ*F*, was 20% of the 1 kHz standard (200 Hz). The test frequency then varied in steps of 40 Hz until the seventh reversal, in steps of 10 Hz for a further four reversals, and in steps of 2 Hz thereafter. Step sizes were attenuated where necessary to prevent Δ*F* < 0. Trial-by-trial feedback was presented visually for 500 ms after each response. The 70.7% frequency discrimination limen [FDL] was computed by averaging over the last eight reversals.

For analysis, the first and last three blocks of data were combined, and the central forth block omitted. This aggregation was necessary in order to provide sufficient data for the nonstationary bias analyses, though it may have caused changes in performance and bias to be underestimated. Performance was measured as mean FDL, averaged across runs. Nonstationary bias was calculated using the presponse-conditional analysis detailed in Experiment II, with two modifications to account for the nonidentical stimuli. First, since relatively few incorrect responses occurred (i.e., as observers were no longer performing at chance), bias was measured following runs of correct presponses only. Second, trials were subdivided by signal magnitude, in order to examine how bias varied as a function of task difficulty.

Two participants were excluded from all analyses because they exhibited significantly poorer thresholds than the average [*p* < .001; FDLs >10 Hz throughout], and so could not provide any estimates of bias at several signal magnitudes. They did, however, exhibit the same basic pattern of reduced FDL (−63.4, −20.2) and reduced bias (–Δ0.14*N*, –Δ0.09*N*).

### Results

#### Learning

Significant learning was observed (Figure 5A), with mean FDL improving from 6.4 to 3.5 Hz [*t*_9_ = 3.14, *p* = .012].[Fig-anchor fig5]

#### Bias

A 3-way repeated measures ANOVA was used to assess how bias varied as a function of *N* presponses, session, and signal level (Figure 5B). As in Experiment II, bias magnitude, *c*, increased with the numbers of identical presponses [*F*_(3,27)_ = 11.13, *p* < .001]. However, both bias magnitude [*F*_(1,9)_ = 9.56, *p* = .013] and the rate at which it increased with *N* [*F*_(3,27)_ = 3.29, *p* = .036] decreased across sessions. This indicates that observers’ nonstationary bias decreased with practice. Post hoc tests indicated that bias no longer increased significantly with *N* presponses in the second half of the experiment [*F*_(3,27)_ = 1.59, *p* = .193, *n*.*s*.], though from inspection of Figure 5B it is clear that some nonstationary bias was present even after practice, even at *N* = 1.

Bias magnitude differed across signal level [*F*_(3,27)_ = 15.22, *p* < .001]. However, there was no straightforward relationship between bias and difficulty, and bias was present even when the stimuli were suprathreshold and ought to have been easily discriminable [6–8 Hz: *F*_(3,27)_ = 6.99, *p* = .001].

There was a strong correlation between changes in threshold, and changes in the rate at which bias increased as a function of *N* [*r*_9_ = 0.87, *p* = .001]. This suggests that a reduction in bias may have contributed to the observed learning effect.

### Discussion

The reported data demonstrated that nonstationary bias decreases with practice. The pattern of response dependencies in Experiment II was replicated in naïve observers, but was attenuated in the latter half of testing. In naïve observers, levels of bias were roughly comparable to those in Experiment II, and were present even at suprathreshold signal levels. This indicates that the nonstationary bias observed in Experiment II was not simply an artifact of the (impossible) task, though we cannot rule out the possibility that response bias is modulated by the observer’s perception of task difficulty.

As in Experiment I, improvements in bias were correlated with improvements in performance, indicating that some perceptual learning may be due to a reduction in nonstationary bias. The precise relationship between bias and performance is explored further below.

#### Simulations: Relating bias to performance

In Experiment I, group-mean *stationary* bias decreased from *c* = 0.31 to *c* = 0.01. In Experiment III, group-mean nonstationary bias decreased from *c* = 0.60*N*, to *c* = 0.19*N* (where *N* is the number of successive, identical presponses). How significant are these changes in terms of the observed changes in performance? To answer this question, correction-factors were derived with which “true” (i.e., unbiased) performance could be recovered. These corrections were then applied to the session-by-session performance/bias estimates in Experiments I and III. To the extent that changes in threshold *decrease* after correcting for bias, perceptual learning can be said to reflect a change in bias. To the extent that changes in threshold increase after correction, perceptual learning can be said to have involved a greater increase in sensitivity than would otherwise be apparent. Here we note evidence of both.

In Experiment I, psychometric functions were fitted to data collected via Method of Constant Stimuli, and stationary bias was measured. In these circumstances, the effect of bias is to shift the psychometric function laterally (Figure 6A), and the necessary bias-correction can be derived analytically. For a cumulative Gaussian psychometric function, the requisite correction is:

$$DL_{corrected}=DL-c\sigma ,$$ 5

[Fig-anchor fig6]

where DL is the estimated detection limen, *c* is estimated bias (in *d′* units; see Equation 1), and σ is the estimated standard deviation of the psychometric function. When this correction was applied to the data from Experiment I, the practice-induced change in threshold increased by 52% (i.e., learning appeared greater after correcting for bias). This increase was largely due to hit rates being overestimated in Session 1, because of observers’ initial bias toward responding “yes”. Thus, the effect of the stationary bias in Experiment I was to cause the true change in sensitivity to be underestimated.

In Experiment III, discrimination limens were derived by averaging reversals on adaptive staircases, and nonstationary bias was measured. In this situation, the necessary correction for bias is not obvious, and may depend on the precise parameters of the adaptive procedure. The required correction factor was therefore determined computationally, using Monte Carlo simulations. In short, performance was simulated given various combinations of sensitivity (i.e., internal noise) and bias. Estimates of performance, DL, and bias, *c*, were then derived, and a bivariate function was fitted that best predicted true DLs given estimated levels of DL and bias (see Section S4 in the online Supplemental Material for details). The result is shown in Figure 6B, and indicated that the necessary correction for bias is well approximated (*r*^2^ > 0.99) by:

$$DL_{corrected}=\Delta_{c}+DL-\frac{\Delta_{c}DL}{2},$$ 6

where Δ_*c*_ is the estimated average rate at which bias, *c*, increased after successive, identical presponses (Figure 3; bottom panels). When this correction was applied to the data from Experiment III, the apparent change in sensitivity decreased by 13%. That is, approximately 0.65 Hz of the 4.9 Hz group-mean improvement was accounted for by changes in bias alone. The remaining learning may be due to changes in sensitivity, or reductions in other forms of bias not measured (e.g., more complex response dependencies). Thus, a substantial minority of the learning in Experiment III was due to reductions in bias.

## General Discussion and Conclusions

This study investigated whether bias is reduced by practice, and the importance of any such changes for our understanding of perceptual learning. In untrained observers, bias was shown to be present in both yes/no and forced-choice tasks. On a yes/no detection task, observers exhibited a stationary bias in favor of responding “yes” (liberal). On 2AFC discrimination tasks, observers were biased by the events of previous trials: perseverating after correct responses and alternating after incorrect responses. Both forms of bias were reduced through practice. Stationary bias was negligible after 1,200 trials, while nonstationary bias was attenuated, but was still present after several thousand trials. The changes in bias meant that practice-induced changes in sensitivity were liable to be underestimated in Experiment I (−52%), and overestimated in Experiment III (+13%). It appears, therefore, that a substantial minority of perceptual learning represents observers learning to ignore previous trials, and to predicate their responses solely on the current sensory information.

### Limitations and Further Considerations

Bias magnitudes, and any changes in bias with practice, may have been underestimated in the present work. This is the case for two reasons. First, because bias tends to be underestimated when samples are relatively small, as was the case in Experiments II and III (see Section S2 in the online Supplemental Material). And second, because in the present work we measured only a small subset of potential biases. Thus, in this case of nonstationary bias, the present work only examined how observers shifted their criterion after repeated correct/incorrect responses. Other trial-sequences may also encourage observers to favor a particular response (e.g., “ABAB,” or “AABB”), and such tendencies may similarly be reduced by practice. Furthermore, observers may exhibit dependencies that extend over a longer range than those studied here. For example, their baseline preference for or against a particular response may undulate throughout the course of the experiment, and this too may be attenuated through practice. The values reported in the present work should therefore be considered only lower bounds, and may increase once other forms of bias are accounted for.

**Figure 1 fig1:**
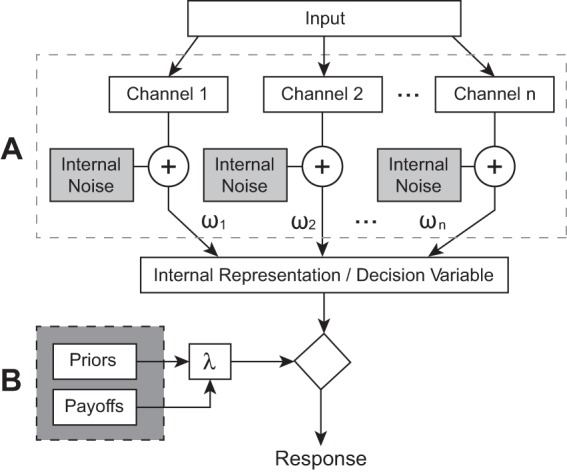
A simple signal detection theory model of decision making (Green & Swets, 1974), adapted from Amitay, Zhang, Jones, and Moore, 2014. (A) The incoming physical stimulus is transformed into an internal representation by summing over *n* information channels, each subject to additive internal noise (the final decision variable may then be further corrupted by late sources of internal noise, not shown here for simplicity). (B) A decision is made by comparing the resultant decision variable to a criterion, λ, which may or may not be optimally placed. Sensitivity is limited by the amount of internal noise, and the observer’s ability to attend selectivity to the task-relevant information channels. Bias is limited by the placement of λ, which may be affected by a range of factors, such as the perceived likelihood of a certain response, or the perceived utility of a certain outcome (see General Discussion). This model is similar to those used in a wide range of papers, both within the perceptual learning literature (e.g., Liu, Dosher, & Lu, 2014; Jones, Moore, Shub, & Amitay, 2014), and more generally (Richards & Zhu, 1994; Tyler & Chen, 2000). Mathematically, this model could be formulated as: respond “yes” if $\left[\sum_{i=1}^{n}\omega_{i}\left(S_{i}+N_{i}\right)\right]$ > λ, otherwise respond “no” (where *S*_*i*_ is the output of the *i*th information channel, and *N*_*i*_ is a corresponding noise sample).

A separate issue is that the present work only examined learning when feedback was provided (supervised learning). The effects of feedback on perceptual learning have been well documented (see Dosher & Lu, 2009; Liu, Dosher, & Lu, 2014). For example, in the absence of feedback, learning may be slowed or even, in some observers, abolished (Herzog & Fahle, 1997; Liu, Lu, & Dosher, 2010). Similarly, there is evidence that both stationary (Petrov, Dosher, & Lu, 2006) and nonstationary (Mori & Ward, 1995; Matthews & Stewart, 2009) bias is attenuated when feedback is presented.^2^ In light of the present results it remains an interesting and open question how feedback moderates the relationship between learning and bias. Thus, bias reduction may be unchanged when feedback is withheld, in which case it would play a proportionally greater role in learning (relative to changes in sensitivity). Conversely, it may be that feedback is required to reduce bias, and that the decreased learning in the absence of feedback in part reflects bias remaining invariant in such circumstances. Although we currently have no data with which to test these two hypotheses, the latter interpretation is consistent with a recent computational model of learning by Liu et al. (2014). Therein, the rate at which bias is attenuated was proportional to perceived accuracy, which was regulated in turn by supervised feedback. It is also interesting to note that the bias in Liu et al.’s (2014) model arises from bottom-up, perceptual mechanisms, and top-down shifts in response criterion are used to compensate for these biases. By contrast, in signal detection theory it is traditionally assumed that the causes of bias arise later in the decision processes [Figure 1], after the sensory information has already been encoded (see below).

### Causes of Bias

**Figure 2 fig2:**
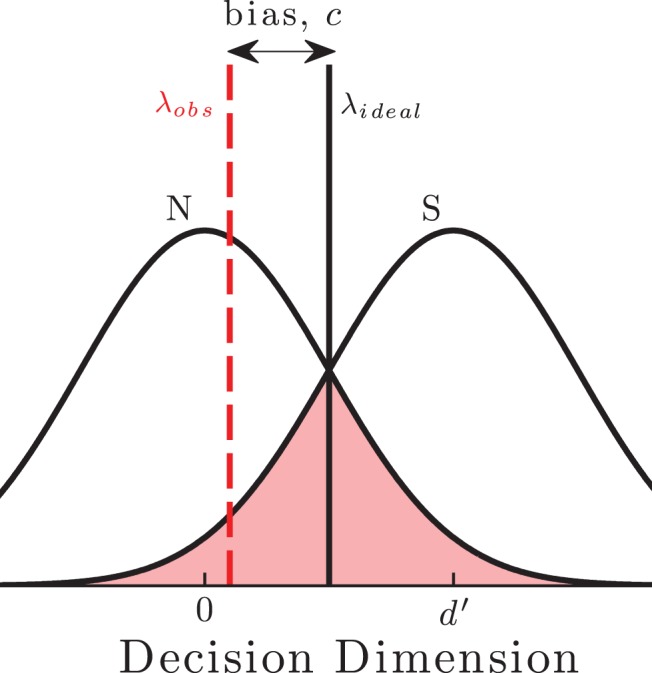
Bias is the distance between the observer’s criterion location, λ_*obs*_ (red [dark gray] dashed), and the ideal criterion location, λ_*ideal*_ (black solid). When noise (*N*) and signal (S) distributions have equal variance (and are sampled from with equal frequency), λ_*ideal*_ is located halfway between their means, as shown here. Here, the observer is overly liberal (biased toward indicating that a signal was present). Performance is also limited by the observer’s sensitivity (or *signal-to-noise ratio*), which is inversely proportional to the common area under the two distributions (highlighted in red [dark gray]). (*N*.*B.* the decision dimension is unspecified, but is typically proportional to some physical aspect of the stimulus, such as its intensity.) See the online article for the color version of this figure.

**Figure 3 fig3:**
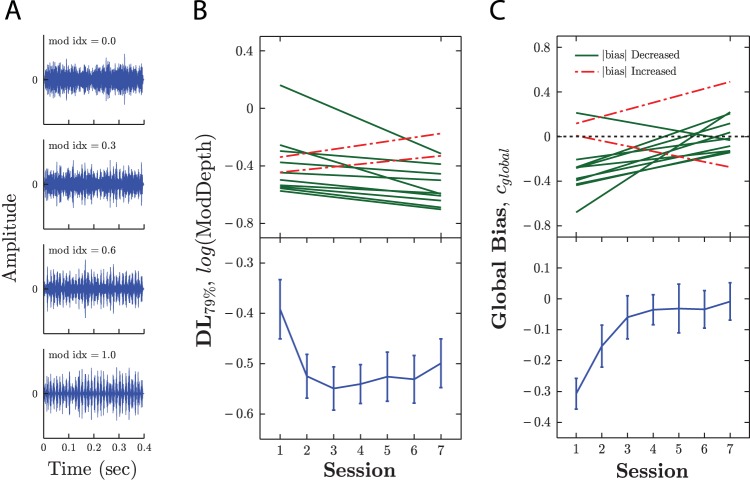
Experiment I: (A) Example stimuli. Showing a range of modulation depths, from zero (top) to full (bottom) modulation. (B) Learning. Group-mean ± 1 *SE* (bottom) detection limens as a function of session, and individual values (top) for first/last session. Individual improvements/decrements in threshold are shown by solid-green [light gray] and dashed-red [dark gray] lines, respectively. (C) Changes in global bias. Group-mean ± 1 *SE* (bottom) global bias (cf. Equation 2) as a function of session, and individual values (top) for first/last session. Individual improvements/decrements in bias magnitude are shown by solid-green [light gray] and dashed-red [dark gray] lines, respectively. See the online article for the color version of this figure.

**Figure 4 fig4:**
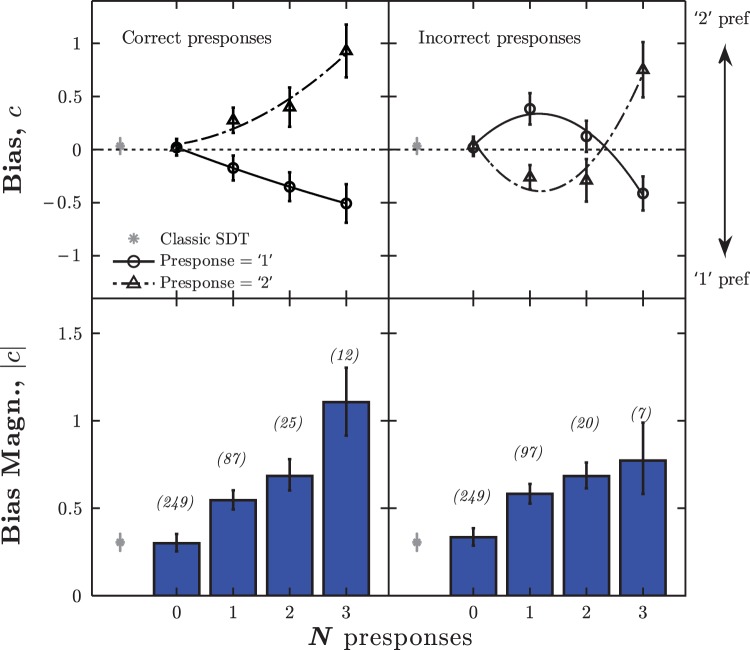
Experiment II: Group mean (±1 *SE*) bias as a function of *N* identical presponses. The left column shows data for identical, correct presponses. The right column shows data for identical, incorrect presponses. The upper row shows signed *c* values (Equation 3) for Interval 1 (solid, circles) and Interval 2 (dashed, triangles) presponses. The lower row shows absolute bias magnitude, |*c*|, averaged across presponse identities. The numbers in parentheses give the mean number of observations (averaged over intervals and observers). The gray marker (far left) shows bias as estimated using all trials, as per classic SDT. Curves represent least-square 2nd-degree polynomial fits. See the online article for the color version of this figure.

**Figure 5 fig5:**
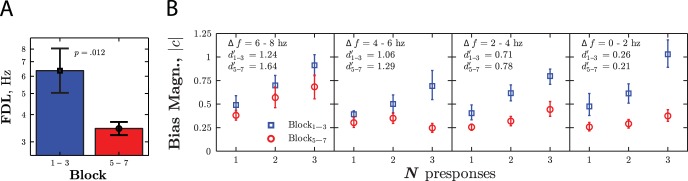
Experiment III: Group mean ± 1 *SE* (A) Sensitivity and (B) Bias magnitude, before (blue [black], squares) and after (red [dark gray], circles) practice. Sensitivity was indexed by the 70.7% frequency discrimination limen. Bias magnitude was measured in the same way as in Figure 4, and was measured independently depending on the *N* presponses (abscissa) and the frequency difference between standard and comparison (panels). See the online article for the color version of this figure.

**Figure 6 fig6:**
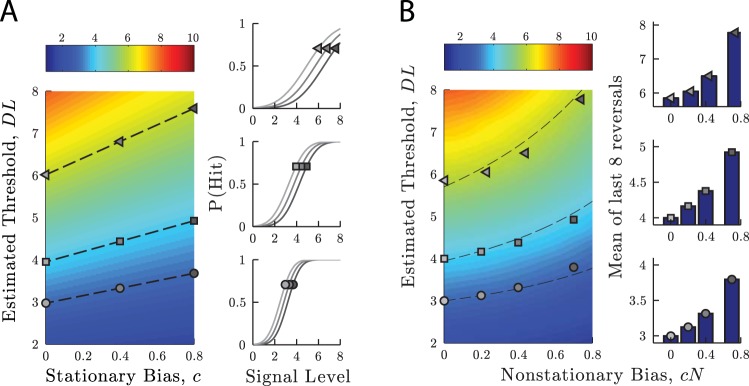
Simulations: Changes in estimated threshold given varying levels of: (A) stationary bias, (B) nonstationary bias. True threshold is indicated by heatmap color. Markers show estimated thresholds at three levels of bias, given a low (circle), medium (square) or high (triangles) true threshold. Dashed lines show the predicted change in estimated threshold, using the correction factors given in Equation 5 (A) or Equation 6 (B). See the online article for the color version of this figure.

The cause(s) of bias in naïve observers remain uncertain. With stationary bias, one possibility is that the asymmetry in response preference reflects a corresponding asymmetry in how observers perceive the statistics of the task. Thus, observers may believe that one response alternative occurs more frequently or yields greater reward, in which case they may shift their criterion so as to favor that response. This could be examined explicitly, by asking observers to rate the relative frequency and utility of each outcome, or implicitly, by asking observers to choose between various lotteries (e.g., by manipulating the relative reward of each response alternative until the observer responds at chance; see Wu, Delgado, & Maloney, 2009). Alternatively, stationary bias may stem from naïve observers misestimating the statistics inherent in their own decision process. For example, random perturbations in an observer’s internal response to a sensory input mean that a signal may be perceived even when none is present. If a naïve observer were unaware of this fact (i.e., if they underestimated their own internal noise magnitude), then the rational response may also be to additively shift their response criterion. For example, an otherwise ideal observer that underestimated their internal noise in Experiment I would have exhibited a liberal bias (as shown graphically in Figure 2). Notably, small samples sizes typically lead to the standard deviation of a normally distributed variable being underestimated. It may therefore be that naïve listeners have insufficient information to accurately estimate their own internal noise levels, and only acquire this information with practice.

The causes of nonstationary bias are potentially more complicated, and may include normative, perceptual and statistical factors. First, in terms of demand characteristics: some observers may believe, for example, that repeating the same response will give the impression that they are being inattentive, uncooperative, or are otherwise malingering. This could explain why observers alternated after incorrect responses, but does not provide an intuitive account of perseverant behavior following correct responses.

Second, biased behavior may emerge from perceptual mechanisms relating to attention. For example, both physiological (Degerman, Rinne, Salmi, Salonen, & Alho, 2006; Alho, Teder, Lavikainen, & Näätänen, 1994; Hesselmann, Sadaghiani, Friston, & Kleinschmidt, 2010) and psychophysical (Demany, Montandon, & Semal, 2004; Hawkins et al., 1990) data suggest that signal gain is heightened in attended regions. If observers paid greater attention to the target interval from the previous trial, then the corresponding stimulus in the following trial may appear greater in magnitude. This would manifest as perseveration when the observer was previously correct (enhancement in same interval), and alternation when previously incorrect (enhancement in alternate interval). Notably, such an explanation predicts that if the task instructions were inverted (“select the quieter or lower-pitched tone”), then the pattern of response-dependencies would be reversed (alternations after correct responses, perseveration after incorrect responses).

Third, response-dependencies may reflect a genuine belief that trials are autocorrelated, such that the target interval in trial *t* is related to the target interval in *t* – 1 (The Gambler’s fallacy: ρ_(*t*, *t* – 1)_≠ 0). This misapprehension of the task statistics may occur if, for example, observers assume (in some cases correctly; e.g., Lindman & Edwards, 1961) that stimuli are being drawn without replacement from a balanced set, such that the occurrence of A makes the subsequent occurrence of B more likely. Alternatively, an assumption of autocorrelation may result from peoples’ tendency to underestimate expected run lengths in a Bernoulli sequence. Thus, even runs of moderate length may appear remarkable, and may be taken as positive evidence that the target interval is correlated across trials. However, perhaps the most parsimonious explanation for why observers may assume a degree of autocorrelation is because in many real-world scenarios this assumption is correct. A given course of action often will yield the same outcome when repeated, and once an action ceases to yield positive results the ideal strategy often is to switch. It is only in context where events are independently distributed (e.g., psychophysical experiments or casinos) that this strategy ceases to be ideal and begins to be considered bias. In this light, the tendency for inexperienced observers to repeat actions that yielded positive outcomes, and avoid responses that yielded negative outcomes, appears quite rational, and it is unsurprising that observers require training to recalibrate their expectations.

We know of no conclusive reasons to favor any of these potential explanations, and it is possible that several factors may operate concurrently. Notably though, all of these considerations—normative, perceptual and statistical—are largely unrelated to the specific tasks of the present study. We therefore predict that the present bias effects should generalize across tasks and modalities.

It is also worth noting that the causes of bias reduction also remain unclear. If bias arises primarily from statistical considerations then it may be that observers are gradually learning the statistics of the task. If this were the case then observers should become more biased if trial-by-trial contingencies are present during training. Alternatively, it may be that bias decreased because observers became fatigued, disengaged, or otherwise ceased trying to draw associations between successive trials (i.e., which in the present tasks happened to be the optimal strategy, but would not be so if a priori trial dependencies existed). If this were the case then bias should decrease even if trial-by-trial contingencies exist during training. Furthermore, one might therefore expect bias to decrease during a session, but peak at the start of each session or following appropriate motivation (e.g., the sudden introduction of a monetary incentive).

### Implications for Perceptual Learning

That perceptual learning involves a reduction in bias suggests that it is, at least in part, a high-order, “cognitive” process. This stands in apparent contrast to the traditional view that perceptual learning is entirely “sensory” and/or peripheral. Historically, this view has been supported principally by the specificity of perceptual learning. For example, practice-induced improvements in performance have been found not to generalize across a particular temporal interval (Karmarkar & Buonomano, 2003; Wright, Buonomano, Mahncke, & Merzenich, 1997) or visual orientation (Fahle & Edelman, 1993; Fiorentini & Berardi, 1980; Karni & Sagi, 1991). That changes in bias would be specific to the stimulus parameters is unintuitive, and constitutes a potential counterexample to the present work. Notably, however, there exists a growing body of evidence that not all learning is stimulus specific, and that some of what is learned does generalize across stimulus parameters (e.g., see Wright & Zhang, 2009). For example, frequency discrimination training at one frequency induces similar, though smaller, improvements in other spectral regions (Irvine, Martin, Klimkeit, & Smith, 2000; Demany, 1985; Amitay, Irwin, & Moore, 2006), while Jeter, Dosher, Liu, and Lu (2010) reported analogous results for a visual orientation discrimination task. It is interesting that in the study by Jeter et al. (2010), the proportion of transfer was greatest in observers who had trained least (1,248 trials). This timescale is consistent with the timescale for bias-reduction reported both here and in the gambler’s fallacy literature (e.g., Ayton & Fischer, 2004; Anderson, 1960; Jarvik, 1951). Furthermore, this timescale is also—as Jeter et al. (2010) note—consistent with the early, rapid stage of perceptual learning (Hawkey, Amitay, & Moore, 2004; Poggio, Fahle, & Edelman, 1992). Thus, it may be that perceptual learning is constituted by multiple mechanisms of differing temporal dynamics. Early learning may be fast, generalizable, and primarily involve observers learning to predicate their decisions solely on sensory information. Later learning may be more gradual, protracted, stimulus-specific, and may primarily concern the efficiency with which sensory information is extracted and processed (potentially via physiological changes in primary-sensory networks).

That learning involves higher-order processes may also help to elucidate some otherwise puzzling phenomena, such as why observers with greater working memory often exhibited enhanced sensory thresholds (e.g., Ahissar & Hochstein, 1997). Thus, if bias relates to observers’ ability to model the task statistics, then observers with greater memory spans may be able to accurately integrate information over more trials. Such observers would be less prone to be misled by local vagaries in the task statistics, such as short runs of identical trials, and so would tend to be less biased. Consistent with this, Barron and Leider (2010) found that observers were biased when attempting to predict the outcome of a virtual roulette wheel, but that this bias was attenuated when the previous 10 outcomes were displayed for review prior to every decision.

### General Implications

The present findings also have a number of implications for psychophysical research more generally. For researchers seeking to avoid the confounding effects of bias, the present results are encouraging. Although observers exhibited substantial bias, even when using supposedly “bias-free” forced-choice methods, these effects were greatly attenuated by practice. Stationary, yes/no bias was eradicated after 1,200 trials, and nonstationary bias was substantially reduced over a similar timeframe. This suggests that one or two practice sessions can largely remove the confounding effects of bias. Moreover, if there are sufficient data with which to estimate bias, corrections were presented here with which true estimates of sensitivity can be recovered.

In some cohorts, however, neither of these approaches may be feasible. For example, when dealing with clinical or developmental populations there is often not time for extensive practice sessions, and small datasets preclude the quantification of bias. In such populations, bias may be causing perceptual sensitivity to be incorrectly estimated, and it may be necessary to develop test protocols that encourage observers to treat each trial independently.

Finally, it may be interesting to consider whether bias varies between populations. For example, children appear, anecdotally, to be highly influenced by the events of preceding trials, and even static interval biases are often inflated in younger observers (e.g., Werner, Marean, Halpin, Spetner, & Gillenwater, 1992; Trehub, Schneider, Thorpe, & Judge, 1991, though see Werner & Marean, 1991). It may therefore be that some group differences (e.g., developmental differences between younger and older listeners) may be in part due to systematic differences in response bias, rather than, as is often assumed, differences in perceptual sensitivity. To answer this question it would be necessary to quantify how response dependencies decrease as a function of age, as well as of experience.

## Conclusions

The principal, novel findings of this work are that:

(1) Bias is present in unpracticed observers, even on forced-choice tasks. On a yes/no task, observers exhibited a stationary bias in favor of responding “yes” (liberal). On a 2AFC task, observers were biased by the events of previous trials (e.g., favoring the previous correct interval), and for a subset of trial sequences these response-dependencies were shown to be cumulative over many (4+) trials. The presence of such bias may lead to perceptual sensitivity being incorrectly estimated in observers naïve to psychophysical testing.

(2) Both stationary and nonstationary bias were reduced through practice. Stationary bias was negligible after 1,200 trials. Nonstationary bias was attenuated by practice, though was not completely eradicated even after several thousand trials.

(3) Simulations indicated that reductions in nonstationary bias accounted for 13% of learning on an 2AFC task. It may be that additional learning is explained by changes in other response dependencies, not measured. Correcting for stationary bias on a yes/no task revealed that sensitivity improved by around 50% more than be would otherwise apparent.

## Supplementary Material

10.1037/xlm0000111.supp

## Figures and Tables

**Table 1 tbl1:** Schema for Selecting Trials Conditional on Repeated, Correct “Interval 2” Presponses, for N = 0 . . . 3

Response	2	2	1	1	2	2	1	2	2	2	1	1	2	1	1	1	2	1
Correct	0	1	1	1	0	1	1	1	1	1	1	0	0	1	1	0	1	1
*N* = 0	†	†		†	†	†		†				†	†	†	†	†	†	
*N* = 1			†				†		†									†
*N* = 2										†								
*N* = 3											†							
																		
*Note.* The first two rows show the target and response intervals for 18 hypothetical trials. For each subset of data, the trials that would be used to calculate bias are marked with an obelisk (†). Analogous subsets of trials (not shown here) were also constructed for those trials preceded by incorrect and/or Interval 1 responses.																		

**Table 2 tbl2:** Percent Correct Responses to Each Interval, and the Resultant Bias Index, c, for N = 0 and N = 1

*N*	Presponse		Target interval		Bias, *c*
	Interval	Correct	1	2	
0	*all*	*all*	48.8	50.9	0.03
	1	no	45.0	56.2	0.14
		yes	58.6	43.5	−0.19
1	2	no	54.1	45.8	−0.11
		yes	37.9	58.0	0.25
*Note.* In the first row all the data is aggregated together (*N* = 0). The near-zero value of *c* indicates minimal bias. In Rows 2–5, the same data is partitioned contingent upon the immediately preceding pesponse (*N* = 1). Positive and negative *c* values indicate Interval 2 and Interval 1 preferences, respectively. The data is a subset of that given graphically in Figure 4.					

## Footnotes

1 Moreover, many studies have demonstrated that observers are capable of integrating over sequential observations when such behavior is beneficial (e.g., Swets, 1959; Berg, 1990).

2 Though it is interesting to note that the no-feedback observers in Herzog and Fahle (1997) actually performed more accurately, initially, than those who were given feedback (see Figure 1 vs. Figure 2 of Herzog & Fahle, 1997). This suggests that in some circumstances feedback may increase bias magnitude.
