# Supplementary material for: The Role of Response Bias in Perceptual Learning
Source: J Exp Psychol Learn Mem Cogn. 2015 Apr 13;41(5):1456–70. doi: 10.1037/xlm0000111 (PMC4562609; doi:10.1037/xlm0000111)
Supplement: Supplementary file 2 [file xlm-XLM-2014-1507-XLM-2014-1507R1_supplemental.pdf]

Supplemental Material: The role of response bias in perceptual learning

§S1: Global bias metric

Formally, bias is given by:

$$bias = \lambda_{obs} - \lambda_{ideal}, \tag{1}$$

where λ_{ideal} is the ideal criterion location, and λ_{obs} is the observer's criterion location, which can be estimated from the observer's false-alarm, f, rates (Wickens, 2002), thus:

$$\widehat{\lambda_{obs}} = Z(1-f) = -Z(f), \tag{2}$$

where Z is the inverse cumulative Gaussian function. With two conditions, if one assumes that the internal responses to both noise and noise+signal are distributed with equal variance (additive internal noise), then $\lambda_{ideal} = \frac{1}{2}d'$. In which case the amount of bias may be indexed by the term c:

$$c = \lambda_{obs} - \frac{1}{2}d' = -\frac{1}{2}[Z(f) + Z(h)], \tag{3}$$

where h is the observed hit rate. More generally the ideal criterion, λ_{ideal}, is that which maximizes the probability of a correct response, P_C. In turn, P_C, is the sum of the probability of a hit and the probability of a correct rejection,

$$P_C = P(hit) + P(correct\ rejection). \tag{4a}$$

In turn, the probability of a hit is the joint probability of a signal trial occurring, P(S), and observer responding 'yes', P('yes'). Likewise, the probability of a correct rejection is the joint probability of a noise trial occurring, P(N), and the observer responding 'no', P('no'):

$$= P(\text{S}, \text{'yes'}) + P(\text{N}, \text{'no'}). \tag{4b}$$

Using the chain rule, this probability can be calculated from the conditional probability of a correct response given that trial type, together with the probability of that trial type occurring:

$$= P(\text{S})P(\text{'yes'} \mid \text{S}) + P(\text{N})P(\text{'no'} \mid \text{N}). \tag{4c}$$

Given a Gaussian detection model, the conditional probability of a correct response can be derived from the cumulative Gaussian distribution, Φ, thresholded at a particular criterion value, λ:

$$= P(\text{S})\Big[1 - \Phi(\lambda; \mu_{signal}, \sigma_{signal})\Big] + P(\text{N})\Big[\Phi(\lambda; \mu_{noise}, \sigma_{noise})\Big]. \tag{4d}$$

For the equal, unit variance model this becomes:

$$= P(\text{S})\Big[1 - \Phi(\lambda; d', 1)\Big] + P(\text{N})\Big[\Phi(\lambda; 0, 1)\Big]. \tag{4e}$$

Finally, when using m signal conditions, this generalises to:

$$= \sum_{i=1}^{m}\Big(P(\text{S})_i\Big[1 - \Phi(\lambda; d'_i, 1)\Big]\Big) + P(\text{N})\Big[\Phi(\lambda; 0, 1)\Big]. \tag{4f}$$

When the observer's goal is to maximize percent correct, the ideal criterion location is therefore given by:

$$\lambda_{ideal} = \arg\max_{\lambda}\left(\sum_{i=1}^{m}\Big(P(\text{S})_i\Big[\Phi(\lambda; d'_i, 1)\Big]\Big) + P(\text{N})\Big[\Phi(\lambda; 0, 1)\Big]\right). \tag{5}$$

And combining Eq 5 with the basic bias formula given in Eq 1 yields:

$$c_{global} = \lambda_{obs} - \arg\max_{\lambda}\left(\sum_{i=1}^{m}\Big(P(\text{S})_i\Big[\Phi(\lambda; d'_i, 1)\Big]\Big) + P(\text{N})\Big[\Phi(\lambda; 0, 1)\Big]\right). \tag{6}$$

The subscript in c_{global} serves to highlight the fact that bias is here computed using a single criterion applied to multiple signals, and to differentiate this, more general measure of bias, from c, which implicitly assumes only one signal+noise distribution.

Note that Eq 5, unlike Eq 3, does not depend on an assumption of equal variance. However, in practice it may be helpful to make this assumption, in which case λ_{obs} and d'_i can again be estimated directly from the hit, h, and false alarm, f, data, thus:

$$c_{global} = -Z(f) - \arg\max_{\lambda}\left(\sum_{i=1}^{m}\Big(P(\text{S})_i\Big[\Phi(\lambda; Z(h_i) - Z(f), 1)\Big]\Big) + P(\text{N})\Big[\Phi(\lambda; 0, 1)\Big]\right). \tag{7}$$

§S2: The sampling distribution of c

Small sample sizes have been shown to statistically bias estimates of sensitivity, d', given a fixed (ideal) criterion, λ. For example, Miller (1996) showed that with small numbers of observations, low values of d' tend to be overestimated,

while high values tend to be underestimated. An analogous statistical bias for estimates of bias, c, could pose a confound for the present study, since the number of samples tended to vary with N presponses. Thus, changes in bias as a function of N presponses may be an artifact of changes in sample sizes, rather than observers shifting their criterion based on previous trials.

To examine how sample size affects the sampling distribution of c, numerical simulations analogous to those of Miller (1996) were run. Monte Carlo estimates of c were made as both d', c, and the number of trials were independently varied. For each combination of values, 10,000 simulations were run, from which the mean and standard deviation of $\hat{c}$ were computed. The results are shown in Fig 1. From individual panels, it can be seen that with small numbers of observations, bias tends to be underestimated. Moreover, moving down the first column, this effect can be seen to interact with the level of bias, with greater levels of bias being underestimated more by small samples. Moving left-to-right across the panels, one also sees how this pattern varies with sensitivity. As d' increases, expected values of bias are increasingly underestimated. Variance in c estimates also tend to decrease as the number of observations increase, and as values of c and/or d' increase.

These findings suggest that statistical bias is unlikely to have qualitatively affected the conclusion that bias increases as a function of N. In fact, they suggest that the effects of bias may have been underestimated, particularly at higher levels of N, where observations were fewer and true values of c appeared to be greater. Thus, levels of bias in naïve observers, and reductions in bias elicited by practice, may be greater than evidenced by the present study.

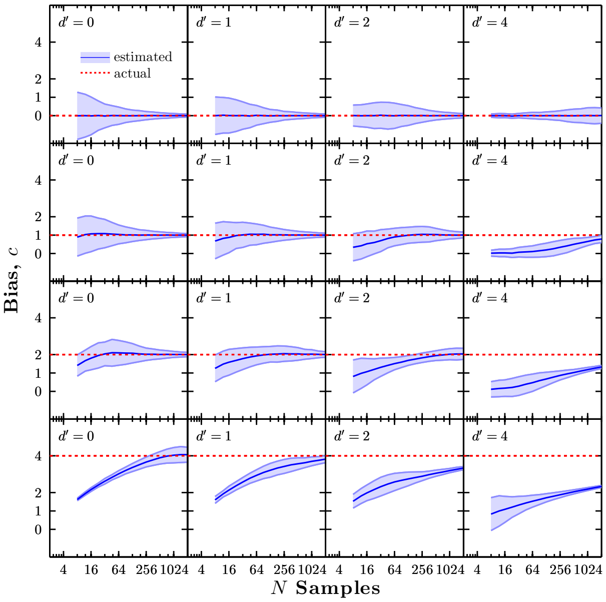

Figure 1. Mean ($\pm$ 1 SD) estimates of bias, $\hat{c}$, as a function of sensitivity (d', columns), true bias (c, rows), and the number of samples (abscissa). True bias is shown by the horizontal dashed red lines. N Samples gives the total number of observations used to compute $\hat{c}$, of which half contained the signal in the first interval.

Presponse		Response Interval							
		Group		Best		Median		Worst	
Interval	Correct	1	2	1	2	1	2	1	2
1	no	1633	2048	10	82	42	69	160	36
	yes	2074	1527	57	37	37	61	152	33
2	no	2044	1728	93	56	71	65	40	17
	yes	1531	2298	26	139	58	96	29	32

Table 1. Number of responses, contingent on presponse identity (interval) and correctness (data from Experiment III). The group data is aggregated over all observers. The Best, Median, and Worst data show individual data, fitted to the idealized group-aggregate response-pattern (see body text).

§S3: χ^2 analyses of sequential dependencies

Chi-square contingency tables were used in Experiment III to further assess response dependencies. Responses were categorized according to the selected interval [1 or 2], the selected presponse interval [1 or 2], and whether or not the presponse was correct [0 or 1]. A chi-square test was used to test whether the 4x2 contingency table of observed values differed significantly from the table of uniformly distributed values that would be predicted by independent trial-by-trial responses.

Naïve observers. The group-aggregate contingency table for Experiment II is given in Table 1. It indicates that the responses of naïve observers were conditional on the immediately preceding trial. Specifically, observers tended to alternate after incorrect presponses, and perseverate after correct presponses. These deviations from a uniform response pattern were significant [$\chi^2(3, 14883) = 303.3, p < .001, V = 0.14$].

At the individual level significant contingencies were also found in 23 of 30 observers [$p < .01$]. These deviations generally followed the same pattern as the group aggregate responses, though to a varying degree. To quantify the similarity between individual observers and the group-aggregate profile, the observed responses of each observer were compared, via the chi-square statistic, with those predicted by an observer who *always* alternated when incorrect and perserverated when correct. For comparison, the values for the best, median and worst fitting individuals are given in Table 1. As per the group-aggregate, the best and median fitting individuals alternated after incorrect presponses, and perseverated after correct presponses. The worst fitting individual exhibited a more general 'Interval 1' preference.

§S4: Simulations

Monte Carlo simulations were used to derive a threshold-correction for nonstationary bias. An ideal observer was simulated, that responded 'Interval 2' if the decision variable (DV) exceeded criterion, $DV > \lambda$, and 'Interval 1' otherwise. The DV was simply the difference in signal magnitude between the two intervals, $S_2 - S_1$, corrupted by an additive internal noise, drawn from a zero-mean, Gaussian distribution: $DV = (S_2 - S_1) + \mathcal{N}(0, \sigma^2)$. On the first trial of each block, and after any response that differed from the preceding, λ was reset to zero (no bias). After every correct response (including those where λ had been reset to zero), λ was cumulatively shifted Δ_c (z-score units) towards the responded interval (i.e., making repetition more likely). Conversely, after every incorrect response, λ was cumulatively shifted Δ_c away from the responded interval (i.e., making alternation more likely). The value of Δ_c therefore corresponds to the mean change in bias as N presponses increases. For each combination of sensitivity (σ: 1 to 12, in 12 uniform steps) and cumulative bias (Δ_c: 0 to 1.2, in 12 uniform steps) 1,000 simulations were run, during which thresholds and bias were calculated in precisely the same way as in Experiment III (e.g., via adaptive tracks, with the same starting value, step sizes, n trials, n reversals, etc.). Increases in threshold, relative to the zero bias condition, were calculated for each simulation, and various predictive functions fitted.

Here we have taken the relatively crude approach of modeling criterion shifts in a purely deterministic manner. In reality, observers probably act more like weighted finite-state automatons (cf. Speeth and Mathews, 1961), whereby a criterion shift is a probabilistic event, and both the magnitude and the relative likelihood (e.g., Thomas et al., 1982) are free parameters. Such an approach would likely yield a picture in which criterion shifts occur less frequently, but with greater effect. However, more complex models such as these would tend to be ill-constrained by perceptual learning datasets, which tend to be small and/or, by definition, unstable.

References

Miller, J. (1996). The sampling distribution of d'. *Attention, Perception, & Psychophysics*, **58**(1), 65–72.

Speeth, S. D. and Mathews, M. V. (1961). Sequential effects in the signal-detection situation. *The Journal of the Acoustical Society of America*, **33**(8), 1046–1054.

Thomas, J., Gille, J., and Barker, R. (1982). Simultaneous visual detection and identification: theory and data. *Journal of the Optical Society of America*, **72**(12), 1642–1651.

Wickens, T. D. (2002). *Elementary signal detection theory*, pages 114–118. Oxford University Press (USA), New York, New York.
